# Fenofibrate attenuates hyperhomocysteinemia-potentiated thrombosis by restoring platelet fatty acid β-oxidation

**DOI:** 10.1016/j.redox.2026.104250

**Published:** 2026-06-06

**Authors:** Lulu Han, Xing Du, Yu Yan, Linqi Zhang, Juan Feng, Xian Wang, Xingzhong Zhang

**Affiliations:** a Cancer Institute, Cellular Therapeutics School of Medicine, Xuzhou Medical University, Xuzhou, China; b Department of Physiology and Pathophysiology, School of Basic Medical Sciences, State Key Laboratory of Vascular Homeostasis and Remodeling, Peking University, Beijing, China; c State Key Laboratory of Cardiovascular Disease, Fuwai Hospital, National Center for Cardiovascular Diseases, Chinese Academy of Medical Sciences and Peking Union Medical College, Beijing, China

**Keywords:** Hyperhomocysteinemia, Platelets, Fatty acid oxidation, Fenofibrate, Thrombosis

## Abstract

Hyperhomocysteinemia (HHcy) is an independent risk factor for thrombotic cardiovascular events. We previously demonstrated that homocysteine (Hcy) amplifies platelet activation by promoting membrane remodeling and enhancing signaling through surface platforms such as integrin αIIbβ3 and G protein-coupled receptors. However, the mechanisms by which Hcy remodels platelet membrane lipid metabolism remain poorly understood. Here, using an integrated proteomic and lipidomic approach, we showed that Hcy disrupted platelet lipid homeostasis by impairing fatty acid β-oxidation (FAO), a metabolic pathway that depends on the coordinated action of peroxisomes and mitochondria. Proteomic profiling showed that Hcy downregulated peroxisome proliferator-activated receptor α (PPARα) and its downstream targets carnitine palmitoyltransferase 1 and 2 (CPT1/2), while lipidomic analysis confirmed the accumulation of medium and long-chain fatty acids, which promoted platelet reactive oxygen species production and mitochondrial dysfunction. Notably, pharmacological activation of PPARα with fenofibrate, a PPARα agonist, restored FAO in a CPT1/2-dependent manner, remodeled the platelet lipid membrane, and attenuated Hcy-potentiated platelet hyperactivation and thrombus formation. Collectively, these findings suggest a previously unrecognized Hcy-PPARα-FAO axis in platelet function and thrombosis, linking impaired peroxisomal and mitochondrial FAO to platelet hyperactivation, and support restoring membrane phospholipid dysregulation as a potential therapeutic strategy for HHcy-promoted thrombotic diseases.

## Highlights

• HHcy suppresses platelet FAO via PPARα/CPT1/2 downregulation.
• Impaired peroxisome-mitochondrial FAO causes lipotoxicity and ROS.
• Fenofibrate restores platelet FAO in a CPT1/2-dependent manner.
• FAO restoration normalizes platelet lipidome and attenuates thrombosis.

## Graphical abstract

**Figure:**
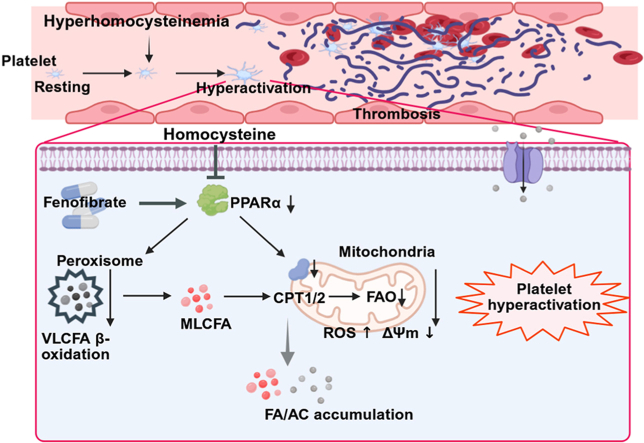

## 1 Introduction

Homocysteine (Hcy) is a sulfur-containing non-proteinogenic amino acid derived from methionine metabolism [1]. Hyperhomocysteinemia (HHcy), defined as plasma Hcy levels exceeding 15 μM, is highly prevalent in Asian populations due to dietary and genetic factors [2,3]. As an independent risk factor for cardiovascular diseases, particularly stroke and acute myocardial infarction, HHcy promotes vascular pathologies such as atherosclerosis and aneurysm by targeting both vascular wall cells (endothelial cells, smooth muscle cells, fibroblasts) and immune cells (macrophages, T cells, B cells) [4,5]. In addition to its effects on vascular and immune cells, accumulating evidence implicates HHcy-potentiated platelet activation and thrombosis as major contributors to cardiovascular events [6,7]. Notably, a large-scale primary prevention study in hypertensive adults demonstrated that baseline platelet count significantly affects the efficacy of folic acid therapy in reducing first stroke risk, suggesting a critical role of platelets in HHcy-related cardiovascular events [3]. These findings collectively underscore the multifaceted role of HHcy in cardiovascular pathogenesis.

Given the central role of platelets in thrombus formation, understanding how Hcy modulates platelet function has garnered considerable attention. Platelets from HHcy individuals exhibit heightened activation status, characterized by increased reactive oxygen species (ROS) production, elevated intracellular Ca^2+^ concentrations, and enhanced thromboxane A2 (TXA2) release [[8], [9], [10], [11]]. Previous studies have elucidated several signaling mechanisms underlying this hyperreactivity [12,13]. For instance, HHcy promotes platelet responsiveness to G protein-coupled receptor (GPCR) agonists such as ADP and thrombin via N-homocysteinylation of β-arrestins, which impairs receptor desensitization and biases GPCR signaling [13]. Our prior work further demonstrated that Hcy enhances integrin αIIbβ3 activation in platelets by promoting phospholipid hydrolysis and facilitating autotaxin binding [12]. Thus, these findings establish that Hcy amplifies platelet activation signals, promoting both platelet activation and thrombus formation.

In platelets, lipids play a triple role: they form the structural backbone of cellular and organelle membranes, act as key signaling mediators, and supply substrates for energy metabolism [14]. Platelet activation triggers rapid membrane lipid remodeling, a process driven by phospholipases such as cPLA2, which couples lipid signaling to the cell's acute energy demands [15]. Notably, sustained energy provision is also essential for preserving phospholipid asymmetry [15], a prerequisite for maintaining membrane integrity and normal platelet function. Together, these roles position fatty acid β-oxidation (FAO) as a central metabolic hub that coordinates lipid remodeling, energy balance, and membrane stability to support effective thrombus formation. This oxidative process is not confined to a single organelle but instead relies on the coordinated action of peroxisomes and mitochondria [16]. Platelets contain these both organelles, though peroxisomes are far less abundant than mitochondria [17,18]. Nevertheless, peroxisomes and mitochondria play distinct yet complementary roles in FAO. Peroxisomes are primarily responsible for the initial chain shortening of very long-chain fatty acids, generating medium- and long-chain fatty acids that cannot be further oxidized within the peroxisomes [19]. This step is catalyzed by peroxisomal β-oxidation enzymes, including acyl-CoA oxidase 1 (ACOX1), acetyl-CoA acyltransferase 1 (ACAA1), and sterol carrier protein 2 (SCP2), while the concomitant production of H_2_O_2_ is neutralized by antioxidant enzymes such as peroxiredoxin 1 (PRDX1), catalase (CAT), and superoxide dismutase 1 (SOD1) [20]. Mitochondria, in contrast, execute the complete oxidation of medium and long-chain fatty acids, as well as those pre-processed by peroxisomes [19]. However, long-chain fatty acids cannot directly traverse the mitochondrial inner membrane. Their entry into the matrix requires conversion to acylcarnitines by carnitine palmitoyltransferase 1 (CPT1), followed by translocation across the inner membrane and regeneration of acyl-CoA by CPT2 [21]. Consequently, impaired FAO leads to the accumulation of acylcarnitines [22,23]. This CPT1/CPT2 axis is thus essential for FAO and has been implicated in platelet activation and function [24]. Despite accumulating evidence linking HHcy to alterations in platelet lipid profiles [12], the precise mechanisms by which Hcy perturbs platelet lipid metabolism—and whether such perturbations contribute to platelet hyperactivation—remain largely unexplored.

This study identifies a functional Hcy-PPARα-FAO axis in platelets that relies on coordinated peroxisome-mitochondria action for efficient FAO. Hcy disrupts this cooperative network, causing lipid accumulation and mitochondrial dysfunction. Conversely, fenofibrate, a PPARα agonist, restores this organelle crosstalk in a CPT1/2-dependent manner, re-establishing platelet lipid homeostasis and attenuating platelet hyperactivation and thrombosis. These results underscore the therapeutic potential of targeting lipid metabolism in HHcy-associated thrombotic diseases.

## 2 Materials and methods

### 2.1 Platelet preparation

Platelets were isolated from C57BL/6J (C57) mice following established methodology detailed in our prior publication [12]. In brief, whole blood was collected via the inferior vena cava from C57 mice (aged 6–8 weeks) under anesthesia induced by 2.5% tribromoethanol (200 mg/kg). To prevent coagulation, the blood was immediately mixed with acid-citrate-dextrose (ACD: containing 2.5% trisodium citrate, 2.0% d-glucose, and 1.5% citric acid) at a 1:7 ratio. Platelet-rich plasma (PRP) was subsequently obtained by centrifuging the anticoagulated blood at 200 g for 11 min. The platelets were then washed two times using CGS buffer (123 mM NaCl, 33 mM d-glucose, 13 mM trisodium citrate, pH 6.5). Following washing, the platelets were resuspended in modified Tyrode's buffer (MTB: 2.5 mM Hepes, 150 mM NaCl, 2.5 mM KCl, 12 mM NaHCO_3_, 5.5 mM d-glucose, 1 mM CaCl_2_, 1 mM MgCl_2_, pH 7.4) at a final concentration of 3 × 10^8^ platelets/mL and allowed to rest at room temperature for 1 - 2 h prior to experimentation.

For *ex vivo* drug treatment experiments, isolated washed platelets were resuspended in MTB and directly incubated with fenofibrate (20 μM), perhexiline (10 μM), acylcarnitine (5-25 μM), or vehicle control, followed by assessments of platelet function, proteomics, or lipidomics as indicated.

### 2.2 Tail bleeding time

In experiments measuring tail bleeding time, the experimental model mice were first anesthetized as describe above. For the assessment, a 5 mm segment was removed from the tail tip and the tail was promptly placed in 37°C isotonic phosphate buffered saline (PBS). The bleeding endpoint was defined as the cessation of blood flow with no recurrence over a subsequent 60-s interval. Complete blood counts and the quantification of hemoglobin (HGB) from tail bleed samples were conducted using a Sysmex XP-100 hematologic analyzer.

### 2.3 Thrombosis model

Platelets were isolated from both control and experimental mice, labeled with the fluorescent dye calcein-AM (Santa Cruz Biotechnology, CA, USA), and subsequently infused intravenously into recipient mice. Following anesthesia, thrombosis was induced by the topical application of 10% FeCl_3_ (Sigma-Aldrich) to mesenteric arterioles or 20% FeCl_3_ to the carotid artery. Thrombus formation was monitored in real time using a fluorescence microscope (Leica Microsystems), with the time to complete vessel occlusion recorded as the experimental endpoint.

### 2.4 HHcy mouse model

To induce chronic HHcy, C57 mice received a normal chow diet and drinking water supplemented with 1.8 g/L homocysteine (DL-Hcy, Sigma, St. Louis, MO, USA) for three weeks, as previously described [12]. Plasma Hcy levels were measured using a commercial ELISA kit (Shanghai Enzyme-linked Biotechnology Co., Ltd., CN). For fenofibrate (Abcam, Cambridge, MA, USA) treatment, mice received oral administration of fenofibrate at a dose of 50 mg/kg every day. Perhexiline (MCE, NJ, USA) was administered daily via oral gavage at 80 mg/kg to inhibit CPT1/2 *in vivo*. Male C57 mice of the wild-type strain, aged 6 - 8 weeks, were sourced from Xuzhou Medical University's Animal Center and housed under standard conditions. All animal procedures were approved by and conducted in compliance with the guidelines of the Experimental Animal Ethics Committee of Xuzhou Medical University, specifically the Laboratory Animal Care and Use Guide.

### 2.5 Acylcarnitine treatment mouse model

Eight-week-old male C57 mice received an intravenous injection of 18:1 acylcarnitine (4 μg in 100 μL saline) for 2 h. Control mice were injected with an equal volume of saline. Platelet function and thrombus formation were then assessed in these animals.

### 2.6 Platelet aggregation

For the *in vitro* Hcy stimulation assay, washed platelets from C57BL/6J mice (3 × 10^8^/mL, 250 μL) were incubated with or without 100 μM Hcy for 10 min at 37°C. In the parallel acylcarnitines stimulation assay, platelets were incubated with or without 5, 10, 15, and 25 μM acylcarnitine 18:1 for 10 min at 37°C. Following these treatments, the platelets were stimulated with thrombin (Sigma-Aldrich, St. Louis, MO, USA), a classical platelet agonist, at the concentrations specified in the figure legends. Platelet aggregation was subsequently recorded using a Chrono-Log lumi-aggregometer at 37°C with constant stirring at 1200 rpm. For inhibition or activation experiments, fenofibrate (20 μM, MCE, NJ, USA), GW6471 (1 μM, MCE, NJ, USA), or vehicle (DMSO, ≤0.1%) was pre-incubated with the platelets for 10 min. Aggregation was monitored continuously for 5-10 min.

### 2.7 Platelet spreading

For the platelet spreading assay, glass coverslips in cell culture plates were coated overnight at 4°C with 10 μg/mL fibrinogen (Sigma-Aldrich) in 0.1 M NaHCO_3_ buffer (pH 8.3). Washed murine platelets were suspended at 2 × 10^7^/mL and seeded onto the coated surfaces. The platelets were allowed to adhere and spread for 2 h at 37°C, stimulated by thrombin either with or without 100 μM Hcy, 20 μM Fenofibrate, or acylcarnitine (5, 10, 15, 25 μM). After incubation, non-adherent cells were removed by washing, and the adherent platelets were fixed, permeabilized, and stained with iFluor-488-conjugated phalloidin (Ouhe Technology, Beijing, CN) to visualize actin filaments. Images were acquired using an Olympus microscope, and the surface area of individual spread platelets was quantified with ImageJ software.

### 2.8 Phosphatidylserine (PS) exposure detection

To assess platelet PS exposure, washed C57 mouse platelets (1 × 10^7^/mL) were stained with an anti-Annexin V antibody (1:40 dilution, Beyotime Biotechnology, NJ, CN) for 20 min at room temperature. The platelets were then incubated at 37°C for 30 min with thrombin (0.01 U/mL), either with or without Hcy (100 μM), fenofibrate (20 μM), or perhexiline (10 μM). After stimulation, the reaction was stopped by adding PBS. Samples were immediately analyzed by flow cytometry using a FACSCalibur instrument (BD Biosciences) or a Cytation 3 Cell Imaging Multi-Mode Reader (BioTek).

### 2.9 Statistical analyses

Statistical analyses were conducted with Prism v.9.0 (GraphPad Software). Data are presented as mean ± SEM unless stated otherwise. An unpaired Student's t-test was used to compare two groups. For comparisons involving multiple groups, we applied one-way ANOVA followed by Tukey's post hoc test. A p-value below 0.05 was considered statistically significant in all analyses.

A complete description of the methods is provided in the supplemental Methods (available on the *Redox Biology* Web site).

## 3 Results

### 3.1 Hcy suppresses platelet FAO and triggers mitochondrial dysfunction

**Fig. 1:**
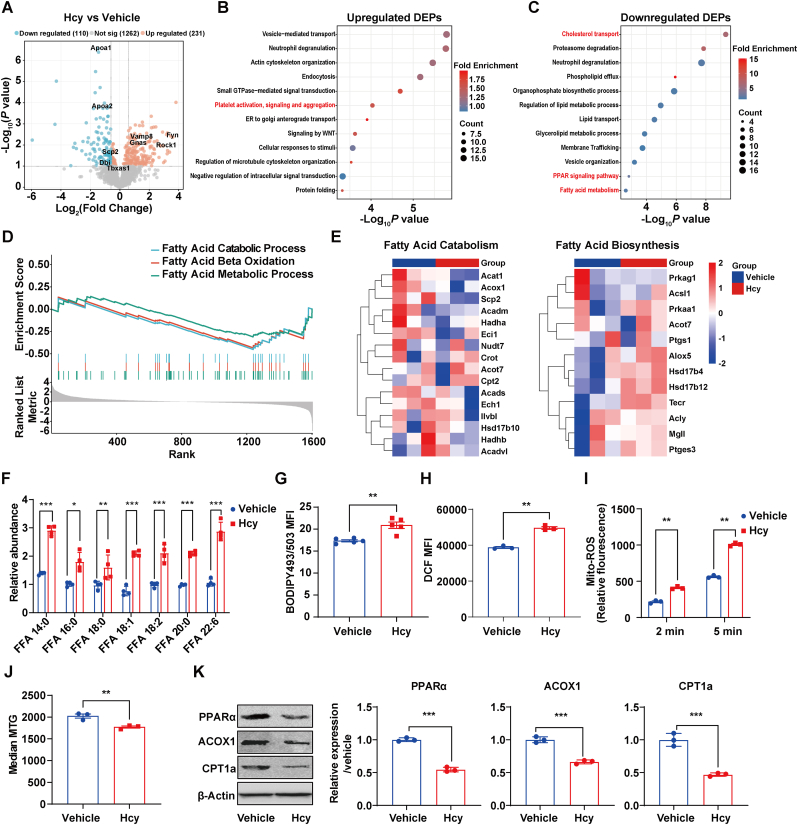
**Hcy suppresses platelet fatty acid β-oxidation (FAO) and triggers mitochondrial dysfunction.** (A) Volcano plot of platelet proteomics after Hcy (100 μM) or vehicle (PBS) treatment for 2 h. DEPs: |log_2_FC| > 0.67, *P* < 0.01 (yellow: upregulated; blue: downregulated; gray: no change). (B, C) GO biological process enrichment of upregulated (B) and downregulated (C) DEPs. (D) GSEA showing enrichment of fatty acid metabolism pathways in Hcy-treated platelets. (E) Heatmap of proteins involved in fatty acid catabolism and biosynthesis. (F) Lipidomics analysis of medium and long-chain fatty acids in Hcy-treated platelets. (G) Neutral lipid accumulation measured by BODIPY 493/503 flow cytometry. (H-J) ROS (H), mitochondrial ROS (I), and mitochondrial membrane potential (ΔΨm) (J) measured by DCFH-DA, MitoSOX, and TMRM by flow cytometry, respectively. (K) Western blot analysis of PPARα, ACOX1, and CPT1a expression. Data are presented as mean ± SEM, n = 3 - 5, **P < 0.01, ***P < 0.001.

To investigate the molecular mechanisms by which Hcy promotes platelet hyperactivation, we first performed an unbiased proteomic profiling of Hcy-exposed platelets. Compared to controls, Hcy treatment resulted in 110 downregulated and 231 upregulated proteins (Fig. 1A). Pathway analysis of these differentially expressed proteins showed enrichment in platelet activation, intracellular signaling, and aggregation, aligning with the pro-thrombotic phenotype (Fig. 1B). GO enrichment analysis further suggested a significant increase in proteins associated with lipid metabolism. Pathways for cholesterol transport, PPARα signaling, and fatty acid metabolism were notably downregulated in Hcy-treated platelets compared to controls. (Fig. 1C). Furthermore, a focused analysis of lipid metabolism-related proteins revealed a general upregulation in pathways associated with fatty acid metabolism in Hcy-treated platelets compared to controls (Fig. 1D). A heatmap visualization further detailed this shift, showing a downregulation of proteins involved in fatty acid catabolism alongside an upregulation of those responsible for fatty acid biosynthesis following Hcy administration in platelets (Fig. 1E).

To determine the effect of Hcy on free fatty acid (FFA) levels in platelets, we performed a lipidomic analysis of platelets exposed to Hcy. The analysis confirmed that Hcy treatment significantly increased medium and long-chain FFA levels (Fig. 1F). Hcy-treated platelets also exhibited elevated neutral lipid content, as demonstrated by BODIPY 493/503 fluorescence staining. (Fig. 1G).

Consistent with the established link between excessive fatty acid oxidation and ROS generation, Hcy-treated platelets exhibited increased total ROS and mitochondrial ROS (mtROS) levels, as measured by DCF and MitoSOX staining, respectively (Fig. 1H and I). JC-1 staining further revealed that Hcy treatment significantly decreased mitochondrial membrane potential (ΔΨm) (Fig. 1J), indicating Hcy-induced mitochondrial dysfunction. In these platelets, the protein expression of key FAO regulators, PPARα, ACOX1, and CPT1a, was also significantly reduced (Fig. 1K). These results suggest that Hcy remodels platelet lipid metabolism by promoting FFA accumulation and biosynthesis while inhibiting FAO, leading to mitochondrial oxidative stress and ultimately mediating platelet hyperactivation.

### 3.2 Activation of FAO by fenofibrate remodels platelet lipid metabolism and alleviates mitochondrial dysfunction

**Fig. 2:**
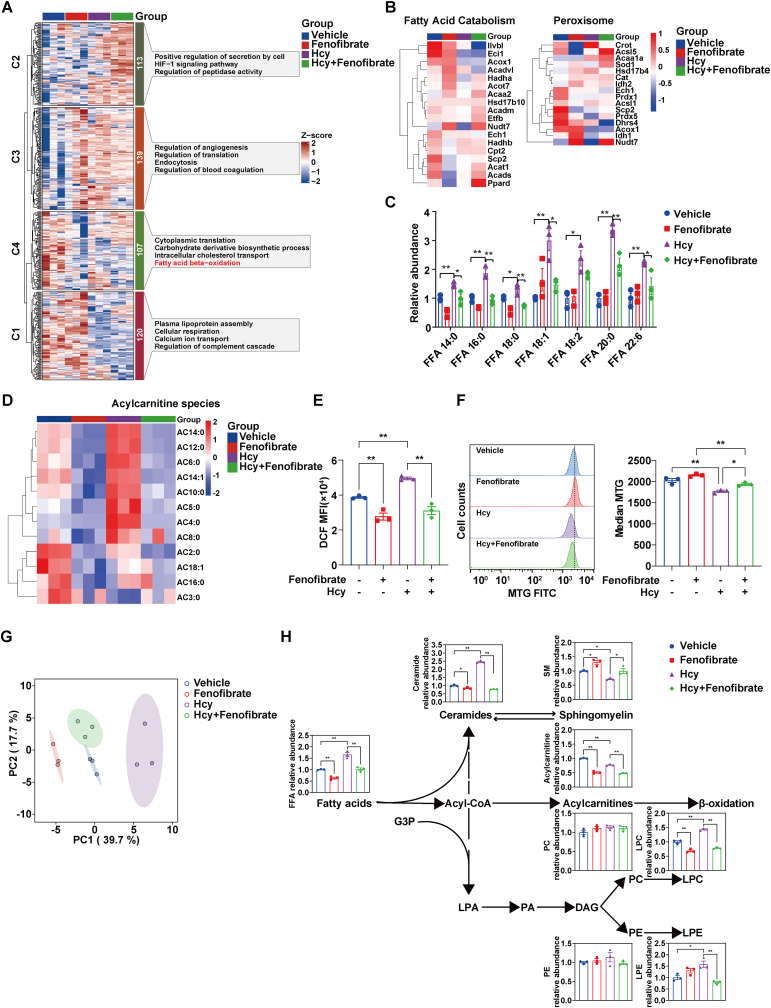
**Activation of FAO by fenofibrate remodels platelet lipid metabolism and alleviates mitochondrial dysfunction.** (A) Platelets were treated with vehicle, fenofibrate (Feno, 20 μM), homocysteine (Hcy, 100 μM), or Hcy + Feno (20 μM). K-means clustering of proteomics profiles with GO enrichment analysis. (B) Heatmap of proteins involved in fatty acid catabolism and peroxisome function. (C) Lipidomics analysis of medium and long-chain fatty acids under the indicated treatments. (D) Heatmap of acylcarnitine (AC) levels. (E, F) ROS levels (E) and mitochondrial membrane potential (ΔΨm) (F) measured by flow cytometry. (G) Principal component analysis (PCA) of proteomics profiles. (H) Schematic of lipid metabolic pathways and quantitative analysis of indicated lipid classes, including ceramide (Cer), sphingomyelin (SM), phosphatidylcholine (PC), phosphatidylethanolamine (PE), lysophosphatidylcholine (LPC), lysophosphatidylethanolamine (LPE), and acylcarnitine. Data are presented as mean ± SEM, n = 3; *P < 0.05, **P < 0.01.

Having established that Hcy suppresses FAO and induces lipid accumulation in platelets, we next investigated whether enhancing FAO could reverse these metabolic abnormalities and mitigate the resulting platelet dysfunction. We therefore employed fenofibrate, a PPARα agonist that promotes FAO [25,26]. To determine if fenofibrate counteracts Hcy-induced platelet hyperactivation by enhancing FAO, we conducted a proteomic analysis of isolated platelets treated *ex vivo* with fenofibrate in the presence or absence of Hcy. The results showed that fenofibrate treatment significantly attenuated Hcy-induced fatty acid accumulation by upregulating pathways involved in fatty acid catabolism and peroxisomal function (Fig. 2A and B), indicating that fenofibrate remodels Hcy-reprogrammed lipid metabolism through activation of FAO. This was confirmed by lipidomic analysis, which suggested that fenofibrate effectively reduced the elevated medium and long-chain fatty acid levels induced by Hcy (Fig. 2C). Consistent with enhanced FAO, acylcarnitine levels, which reflect FAO flux, were significantly decreased after fenofibrate treatment (Fig. 2D). These findings indicate that fenofibrate activates platelet FAO pathway.

Concurrently, we assessed the impact of Hcy on platelet oxidative status and mitochondrial function. Hcy treatment significantly elevated intracellular ROS levels, as indicated by increased DCF fluorescence, and promoted lipid peroxidation, evidenced by elevated BODIPY-C11 signal. Fenofibrate co-treatment effectively mitigated these Hcy-induced increases (Fig. 2E and S Fig. 1A). Furthermore, Hcy diminished mitochondrial membrane potential (ΔΨm), a marker of mitochondrial health, while fenofibrate restored it toward baseline levels (Fig. 2F), suggesting that fenofibrate ameliorates Hcy-induced lipid peroxidation and mitochondrial dysfunction.

To further elucidate whether the protective effect of fenofibrate is linked to remodeling of the lipid profile, we conducted a comprehensive lipidomic analysis. Principal component analysis (PCA) revealed distinct clustering among the four treatment groups. Along the primary component (PC1), Hcy-treated samples exhibited a marked rightward shift relative to the vehicle control, indicating a profound Hcy-induced alteration in the global lipidome. Importantly, samples treated with Hcy plus fenofibrate shifted leftward on the PCA plot, clustering closer to the vehicle group, which suggests that fenofibrate largely normalizes the Hcy-reprogrammed lipid profile (Fig. 2G). Consistent with this, heatmap analysis showed that Hcy stimulation increased ceramides while decreasing their precursor sphingomyelins (SMs) (SFig. 1B). Fenofibrate co-treatment reversed these alterations (SFig. 1B). Similar opposing trends were observed for lysophosphatidylcholine (LPC) and lysophosphatidylethanolamine (LPE) (SFig. 1B). Quantitative analysis confirmed these coherent changes in key lipid metabolic pathways (Fig. 2H). These data indicate that PPARα activation counteracts Hcy-induced lipotoxicity and oxidative stress by activating FAO and restoring phospholipid metabolic homeostasis in platelets.

### 3.3 Fenofibrate ameliorates HHcy-induced platelet hyperactivation and reduces thrombus formation

**Fig. 3:**
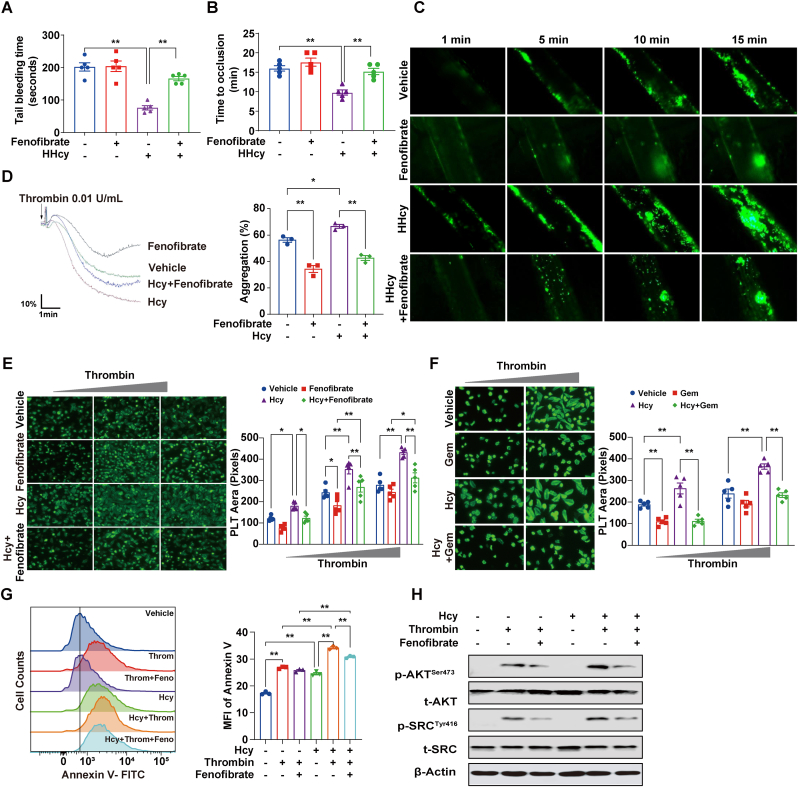
**Fenofibrate ameliorates HHcy-induced platelet hyperactivation and reduces thrombus formation.** (A) Tail bleeding time in mice treated with Hcy (1.8 g/L in drinking water for 3 weeks) ± fenofibrate (Feno, 100 mg/kg, p. o. every other day starting from week 1). (B, C) Occlusion time (B) and representative images (C) of FeCl_3_-induced thrombosis in mesenteric arterioles. (D) Platelet aggregation measured by turbidimetry after treatment with Hcy (100 μM) ± Feno (20 μM). (E, F) Platelet spreading area on fibrinogen after treatment with Hcy (100 μM) ± Feno (20 μM) (E) or ± gemfibrozil (Gem, 50 μM) (F). (G) Phosphatidylserine (PS) exposure assessed using Annexin V staining by flow cytometry (representative histograms and quantification). (H) Western blot analysis of p-AKT^Ser473^ and p-SRC^Tyr416^ in platelets stimulated with thrombin (0.01 U/mL) ± Hcy (100 μM) ± Feno (20 μM). Data are presented as mean ± SEM, n = 5; *P < 0.05, **P < 0.01.

Our data suggested that Hcy suppresses FAO and promotes phospholipid accumulation in platelets, whereas fenofibrate reverses these effects by activating the PPARα/FAO pathway. Therefore, we used the PPARα agonist fenofibrate to test whether stimulating this pathway mitigates Hcy-triggered platelet hyperactivation and thrombosis in the HHcy mice. We found that fenofibrate significantly reversed the shortened tail bleeding time caused by HHcy (Fig. 3A). Fenofibrate also restored the reduced vessel occlusion time and reduced thrombus formation associated with HHcy (Fig. 3B and C). No significant differences were observed in white blood cell count, platelet count, mean platelet volume, platelet distribution width, or plateletcrit across the experimental groups **(**SFig. 2A). Serum homocysteine levels were measured and found to be significantly elevated in the HHcy mice (SFig. 2B). As anticipated, in *ex vivo* treated isolated platelets, fenofibrate markedly suppressed Hcy-induced irreversible platelet aggregation (a hallmark of platelet thrombus formation) and spreading (an integrin αIIbβ3-mediated process critical for thrombus stabilization) (Fig. 3D and E). This effect was corroborated by another PPARα agonist, gemfibrozil, which similarly inhibited the Hcy-mediated increase in platelet spreading *in vitro* (Fig. 3F). In contrast, the potent PPARα antagonist GW6471 exacerbated the increase in platelet aggregation caused by Hcy **(**SFig. 2C). In line with our previous findings that Hcy promotes PS exposure (an event that exposes a procoagulant surface for thrombin generation) and subsequent integrin αIIbβ3 signaling activation [12], fenofibrate treatment significantly attenuated both Hcy-induced PS exposure and the activation of the αIIbβ3 pathway (Fig. 3G and H). These results indicate that fenofibrate alleviates HHcy-induced platelet hyperactivation and thrombus formation.

### 3.4 Acylcarnitine enhances platelet activation and promotes thrombosis

**Fig. 4:**
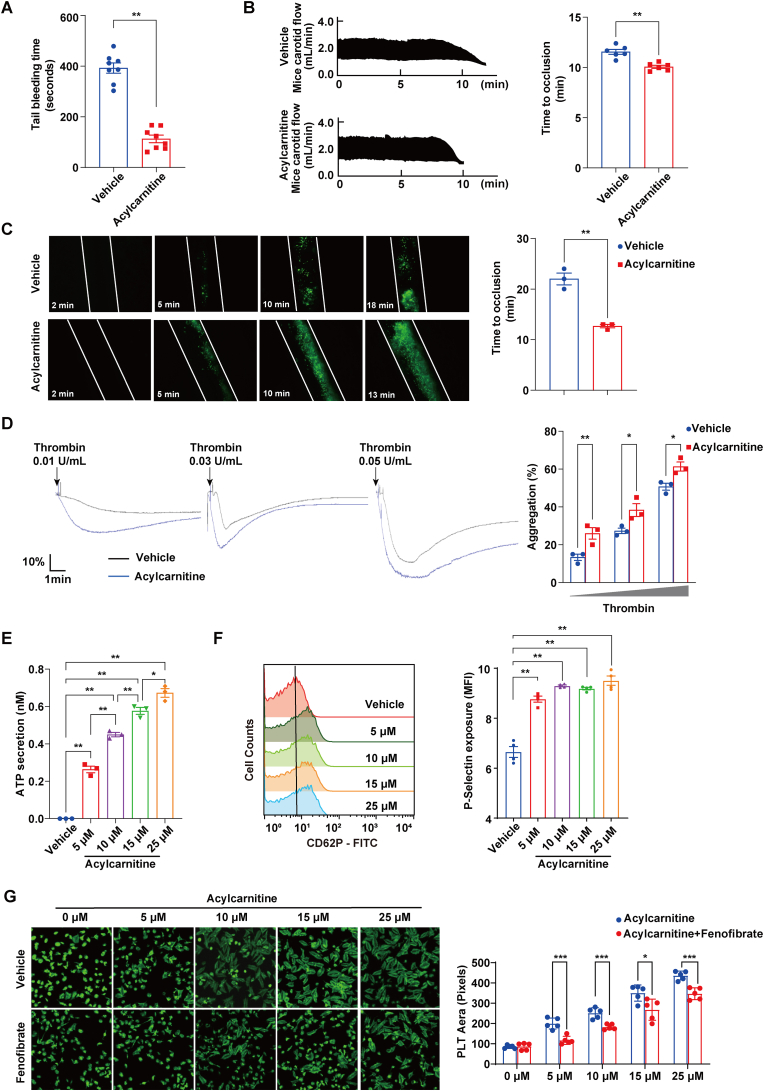
**Acylcarnitine enhances platelet activation and promotes thrombosis.** (A) Tail bleeding times were measured in C57 mice 2 h after an intravenous injection of 18:1 acylcarnitine (4 μg in 100 μL saline). (B) Occlusion times were determined using a FeCl_3_-induced thrombosis model in the carotid artery following acylcarnitine administration. (C) Similarly, occlusion times were assessed in the mesenteric arterioles using the FeCl_3_-induced model. (D) Platelet aggregation was monitored turbidimetrically with 25 μM acylcarnitine in the presence of the indicated thrombin concentrations. (E) ATP secretion was measured in platelets stimulated with the indicated doses of acylcarnitine. (F) P-selectin exposure was quantified in platelets stimulated with the indicated acylcarnitine concentrations. (G) Platelet spreading was assessed following stimulation with the indicated doses of acylcarnitine or fenofibrate. Data are presented as mean ± SEM, n = 3 - 8, *P < 0.05, **P < 0.01, ***P < 0.001.

Accumulation of medium and long-chain acylcarnitines is a recognized driver of mitochondrial damage and platelet dysfunction [22]. Given that Hcy induced acylcarnitine accumulation and that fenofibrate reversed this elevation in Hcy-stimilated platelets, we next investigated whether acylcarnitine itself directly promotes platelet activation and thrombosis. To this end, we administered acylcarnitine (200 μg/kg) via tail vein injection and evaluated platelet function *in vivo*. Acylcarnitine treatment significantly shortened tail bleeding time (Fig. 4A). In FeCl_3_-induced thrombosis models of the carotid artery and mesenteric arterioles, acylcarnitine administration markedly accelerated vessel occlusion (Fig. 4B and C), indicating that acylcarnitine promotes platelet activation and thrombus formation *in vivo*. We then evaluated the direct *in vitro* effects of acylcarnitine on platelet aggregation and activation. Pretreating isolated murine platelets with acylcarnitine (10 μM) *ex vivo* enhanced their aggregation in response to thrombin (Fig. 4D). Acylcarnitine alone, without any classical agonist, directly triggered platelet degranulation in a dose-dependent manner (5 - 25 μM), as shown by increased ATP secretion and P-selectin surface expression—two classic markers that reflect the release of granular contents, which amplifies platelet activation through autocrine and paracrine signaling (Fig. 4E and F). Acylcarnitine stimulation also significantly promoted platelet spreading across the same dose range (5 - 25 μM), and this pro-spreading effect was reversed by co-treatment with fenofibrate (20 μM) (Fig. 4G). These results indicate that acylcarnitine stimulates platelet aggregation, granule secretion, and pro-thrombotic activity. Acylcarnitine thus promotes platelet activation and thrombosis, accelerating vessel occlusion and shortening bleeding time *in vivo* while directly enhancing platelet aggregation, degranulation, and spreading *in vitro*.

### 3.5 Perhexiline, a CPT1/2 antagonist, abolishes fenofibrate's protective effect against HHcy-induced thrombosis

**Fig. 5:**
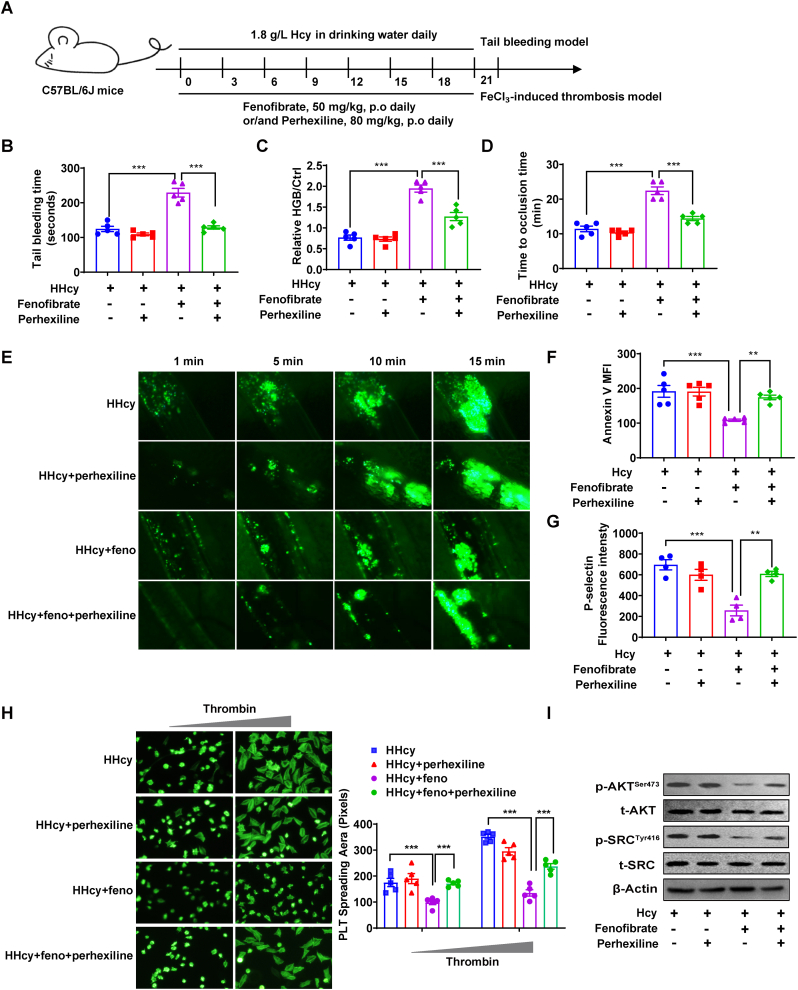
**Perhexiline, a CPT1/2 antagonist, abolishes fenofibrate's protective effect against HHcy-induced thrombosis.** (A) The experimental schematic for the *in vivo* study is shown. (B) Tail bleeding time and (C) blood loss based on hemoglobin (HGB) quantification were measured. (D) Occlusion times were recorded in mesenteric arterioles, and (E) representative images depict thrombi induced by FeCl_3_. (F-G) Platelet function *in vitro* was assessed by measuring PS exposure (F), P-selectin exposure (G) with a Cytation 3 Cell Imaging Multi-Mode Reader. (H) Platelet spreading was assessed following stimulation with thrombin. (I) Western blot analysis determined the levels of p-AKT^Ser473^ and p-SRC^Tyr416^ in platelets treated with Hcy, fenofibrate, or perhexiline. Data are presented as mean ± SEM (n = 4 - 5), *P < 0.05, **P < 0.01, ***P < 0.001.

To determine whether fenofibrate mitigates HHcy-induced platelet activation and thrombosis specifically by enhancing FAO, we co-treated HHcy mice with fenofibrate and perhexiline, an oral inhibitor of CPT1/2 that blocks mitochondrial import of medium and long-chain fatty acids [27] (Fig. 5A). Serum Hcy was significantly elevated in HHcy mice **(**SFig. 3**)**. Fenofibrate significantly reversed the HHcy-induced shortening of tail bleeding time and reduction in blood loss (Fig. 5B and C). It also rescued the HHcy-mediated decrease in occlusion time and increase in thrombus formation (Fig. 5D and E). Co-administration of perhexiline, however, abolished these protective effects, exacerbating both bleeding and thrombotic severity (Fig. 5B–E). These phenotypic response were corroborated *in vitro*. The inhibitory effect of fenofibrate on platelet spreading, P-selectin and PS exposure, and integrin αIIbβ3 signaling (p-AKT^Ser473^ and p-SRC^Tyr416^) were reversed by perhexiline, consequently increasing activity (Fig. 5F–I). These results indicate that fenofibrate protects against HHcy-induced platelet hyperactivation and thrombosis in a CPT1/2-dependent manner.

### 3.6 CPT1/2 inhibition by perhexiline reverses fenofibrate-induced remodeling of platelet FAO and phospholipid metabolism

**Fig. 6:**
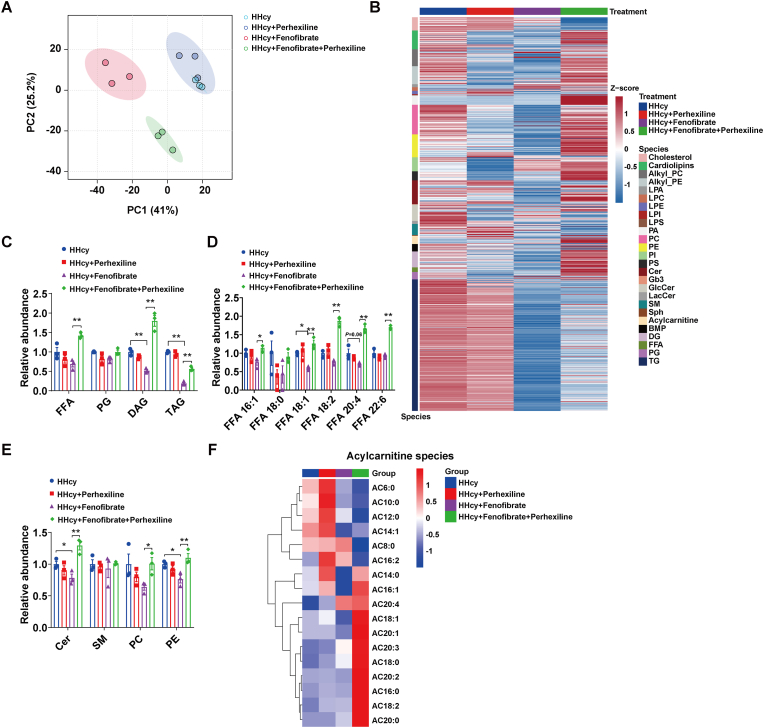
**CPT1/2 inhibition by perhexiline reverses fenofibrate-induced remodeling of platelet FAO and phospholipid metabolism.** Platelets were isolated from the mice in Fig. 5 for lipidomics analysis. (A) Principal component analysis (PCA) of the platelet lipidome, with each point representing an independent biological replicate colored by treatment group. (B) Heatmap displaying the relative abundance of individual lipid species in the platelet lipidomic profile across treatment groups. (C) Bar graph showing the normalized relative total concentration of free fatty acids (FFA), phosphatidylglycerol (PG), diacylglycerol (DAG), and triacylglycerol (TAG). (D) Bar graph showing the normalized relative abundance of FFA across different chain lengths. (E) Bar graph showing the normalized relative total concentration of key phospholipid and sphingolipid classes: ceramide (Cer), sphingomyelin (SM), phosphatidylcholine (PC), and phosphatidylethanolamine (PE). (F) Heatmap displaying the normalized relative levels of individual acylcarnitine (AC) species relative to the HHcy group, with color intensity indicating fold-change (red: upregulation; blue: downregulation). Data are presented as mean ± SEM (n = 3), *P < 0.05, **P < 0.01, ***P < 0.001.

To determine whether fenofibrate's benefit involves CPT1/2-mediated FAO in HHcy, we performed a targeted lipidomic analysis of platelets after administering the CPT1/2 inhibitor perhexiline to the mice in Fig. 5. Principal component analysis (PCA) of the platelet lipidome showed clear separation among the treatment groups (Fig. 6A). The HHcy and HHcy + perhexiline groups clustered together, while the HHcy + fenofibrate and HHcy + fenofibrate + perhexiline groups formed distinct clusters. The HHcy + fenofibrate group displayed a marked leftward shift along the first principal component relative to the HHcy group; this shift was reversed toward the right in the HHcy + fenofibrate + perhexiline group, indicating that perhexiline counteracted the lipidomic remodeling induced by HHcy + fenofibrate.

A heatmap analysis confirmed that fenofibrate co-treatment significantly lowered the levels of triacylglycerols (TAGs), diacylglycerols (DAGs), and medium and long-chain free fatty acids that were elevated by HHcy (Fig. 6B). These changes are consistent with PPARα activation, which stimulates triglyceride breakdown and FAO, thereby supporting the validity of our lipidomics data. Quantitative analysis of total lipid classes verified a significant decrease in TAG, DAG, and total FFA pools following fenofibrate treatment (Fig. 6C). Importantly, this decrease was largely reversed when the CPT1/2 inhibitor perhexiline was co-administered, implying that fenofibrate acts through the CPT1/2 pathway to regulate fatty acid metabolism. The free fatty acid chain-length profile further reinforced this conclusion, showing a fenofibrate-dependent reduction across multiple chain lengths that was attenuated by perhexiline (Fig. 6D). Fenofibrate also significantly altered other key lipid classes, reducing the levels of ceramides and lysophospholipids (LPC and LPE) while increasing phosphatidylcholine (PC) and phosphatidylethanolamine (PE) that were perturbed by HHcy. These fenofibrate-induced changes were largely reversed upon co-treatment with perhexiline (Fig. 6B–E and SFig. 4A–C). Concurrent acylcarnitine profiling suggested that fenofibrate normalized the HHcy-driven accumulation of several long-chain acylcarnitine species, an effect that was similarly blocked by CPT1/2 inhibition (Fig. 6F). Collectively, these lipidomic results demonstrate that fenofibrate corrects the pro-thrombotic lipid profile, characterized by elevated phospholipids and AC, via a CPT1/2-dependent FAO mechanism, which aligns with the attenuation of the thrombotic phenotype shown in Fig. 5.

## 4 Discussion

Here, we provide pharmacological evidence that Hcy suppresses FAO by downregulating PPARα and CPT1/2, leading to phospholipid accumulation and mitochondrial dysfunction. This FAO blockade drives membrane phospholipid remodeling—marked by increased LPC/LPE generation and a shift from SM to ceramide—which amplifies membrane signaling and enhances platelet responsiveness to GPCR agonists such as thrombin. Notably, a PPARα agonist, such as fenofibrate, reverses these effects by restoring FAO in a CPT1/2-dependent manner, thereby normalizing Hcy-induced platelet phospholipid dysmetabolism and attenuating hyperactivation and thrombus formation.

HHcy disrupts glucose and lipid metabolism across multiple organ systems, with growing evidence linking Hcy-induced vascular damage to metabolic reprogramming in immune cells. Our previous work demonstrated that Hcy drives glycolytic reprogramming in T cells, B cells, and macrophages by upregulating PKM2, thereby promoting pro-inflammatory responses and increasing IgG production to accelerate atherosclerosis [[28], [29], [30], [31]]. Beyond its effects on glucose metabolism, Hcy also broadly perturbs lipid metabolism—affecting fatty acid synthesis, uptake, and lipolysis in various cell types [[32], [33], [34]]. Among these diverse lipid metabolic processes, FAO, which relies on the coordinated action of peroxisomes and mitochondria, has emerged as a central pathway susceptible to Hcy-induced dysfunction. Consistent with these broad metabolic effects, our study demonstrates that HHcy disrupts platelet lipid homeostasis by suppressing PPARα and its downstream targets CPT1/2, which impairs mitochondrial FAO and induces lipotoxic stress. These findings establish that HHcy promotes systemic metabolic imbalance by driving glycolytic reprogramming while impairing phospholipid metabolism. This pattern of metabolic dysregulation operates across diverse cell types, including immune cells, platelets, hepatocytes, and adipocytes.

Platelets rely on fatty acid oxidation to meet their energy demands, as fatty acids generate roughly six times more ATP per mole than glucose [35]. Fenofibrate, a PPARα agonist, activates fatty acid oxidation and reduces hypertriglyceridemia [36]. Here, we demonstrate that fenofibrate restores Hcy-impaired mitochondrial FAO, thereby alleviating lipid accumulation, mitochondrial dysfunction, and ROS production. In addition, fenofibrate attenuates HHcy-induced platelet hyperactivity and prothrombotic behavior. A similar protective effect was observed with another PPARα agonist, gemfibrozil, whereas the antagonist GW6471 enhanced platelet aggregation under both basal and HHcy-treated conditions. These results confirm that PPARα activation ameliorates HHcy-potentiated platelet dysfunction, consistent with a previous report showing that omega-6 DPA and its 12-lipoxygenase metabolites inhibit platelet activation and thrombosis in a PPARα-dependent manner [37]. Therefore, these findings suggest that PPARα activation not only improves hypertriglyceridemia but also confers anti-thrombotic benefits, particularly in patients with HHcy [38].

Based on our findings, the protective effects of fenofibrate against HHcy-potentiated platelet hyperactivity were largely abrogated by CPT1/2 inhibition, indicating that CPT1/2, as direct targets of PPARα, play an essential role in fatty acid oxidation. This is consistent with the critical role of the CPT1/2 axis in platelet biology highlighted by Liu and colleagues [24], who showed that reduced CPT2 expression and activity impair FAO and lead to accumulation of long-chain acylcarnitines, thereby contributing to mitochondrial damage in platelets [24]. In our study, Hcy-mediated FAO inhibition similarly triggered acylcarnitine accumulation in platelets. Notably, accumulated acylcarnitine themselves promoted platelet aggregation and thrombus formation in a paracrine/autocrine manner. These observations align with previous studies showing that 2-methylbutyrylcarnitine enhances thrombosis via integrin α2β1 [39], and may help explain why elevated even-chain acylcarnitine (acylcarnitine: C2, C8, C16) correlate with increased cardiovascular mortality [40]. Conversely, fenofibrate reversed HHcy-induced acylcarnitine accumulation in the platelets, underscoring that restoration of FAO is essential for its anti-thrombotic action.

Fenofibrate also remodels the HHcy-distorted lipidome, reversing the elevations in LPC, LPE, and ceramide, as well as the reduction in SM. We previously showed that Hcy activates cPLA2, driving the conversion of PC to LPC and boosting arachidonic acid and TXA2 production—all of which promote platelet activation [12]. Fenofibrate markedly lowers Hcy-induced LPC/LPE levels, indicating that FAO activation modulates membrane phospholipid turnover. These findings further suggest that platelet peroxisomes and mitochondria are indispensable not only for energy homeostasis but also for maintaining membrane phospholipid asymmetry [14], highlighting these pathways as pivotal for preserving platelet membrane integrity and supporting thrombus formation. Fenofibrate also significantly reduces ceramide levels while increasing SM, shifting the sphingolipid balance toward homeostasis. Ceramide is a well-established cardiovascular risk factor that regulates platelet inflammation and function [41]. Ceramide exacerbates atherosclerosis through its receptors CYSLTR2 and P2RY6 [42]. Conversely, alkaline ceramidase 1-mediated platelet ceramide catabolism relieves vascular inflammation [43]. Thus, fenofibrate-mediated restoration of sphingolipid homeostasis may further contribute to its anti-thrombotic effects. Similarly, elevated LPC and LPE levels have been implicated in chronic inflammation and atherosclerosis [44,45], and fenofibrate-induced reduction of these lysophospholipids may represent an additional anti-thrombotic mechanism. Together, our results highlight the coordinated function of peroxisomes and mitochondria in platelet lipid metabolism and support targeted FAO restoration as a promising therapeutic approach for platelet-driven thrombotic disorders.

A limitation of our study is that we relied solely on pharmacological approaches (fenofibrate and gemfibrozil) to activate PPARα, without employing genetic loss- or gain-of-function strategies such as platelet-specific PPARα knockout or overexpression. While the use of the two distinct agonists strengthens the pharmacological evidence, future studies using platelet-specific PPARα-deficient or overexpressing mouse models will be essential to definitively establish the cell-autonomous requirement of PPARα in mediating the protective effects against HHcy-induced platelet dysfunction. Additionally, while we have clarified that platelets contain peroxisomes, the direct contribution of peroxisomes to platelet FAO and Hcy-induced dysfunction was not experimentally addressed in this study. Future investigations using peroxisome-specific interventions are warranted to dissect the relative roles of these two organelles. Furthermore, our findings are derived from mouse models *in vivo, ex vivo* and *in vitro* platelet studies; clinical validation in human subjects is lacking. Although fenofibrate has been shown to reduce atherosclerotic cardiovascular events and stroke [46,47], and HHcy is a well-established risk factor for these conditions, direct evidence for fenofibrate's anti-thrombotic effects specifically in HHcy patients remains to be established. Future clinical studies are warranted to determine whether fenofibrate exerts similar platelet-targeted metabolic benefits in HHcy patients and whether these effects translate to reduced thrombotic risk.

## 5 Conclusion

These findings support the existence of an Hcy-PPARα-FAO axis that links metabolic dysfunction to platelet membrane phospholipid remodeling—central to amplifying prothrombotic signaling. By restoring FAO and normalizing the platelet lipidome, PPARα activation offers a novel strategy to counter HHcy-potentiated platelet hyperactivation and thrombus formation. Collectively, our work positions metabolic intervention in platelets as a promising therapeutic approach for HHcy-associated thrombotic diseases.

## Funding

This work was supported by grants from the 10.13039/501100001809National Natural Science Foundation of China (No. 82070462 to Xian Wang, No. 82203172 to Lulu Han, and No. 82300327 to Xingzhong Zhang).

## CRediT authorship contribution statement

**Lulu Han:** Data curation, Formal analysis, Funding acquisition, Investigation, Methodology, Writing – original draft. **Xing Du:** Data curation, Formal analysis, Investigation, Methodology, Writing – original draft. **Yu Yan:** Data curation, Investigation, Methodology. **Linqi Zhang:** Data curation, Investigation, Methodology. **Juan Feng:** Conceptualization, Project administration, Supervision, Writing – review & editing. **Xian Wang:** Conceptualization, Funding acquisition, Project administration, Resources, Supervision, Writing – review & editing. **Xingzhong Zhang:** Conceptualization, Data curation, Formal analysis, Funding acquisition, Project administration, Supervision, Writing – original draft, Writing – review & editing.

## Declaration of competing interest

I have nothing to declare.

## Data Availability

The proteomics data have been deposited with the ProteomeXchange Consortium via the iProX partner repository under Project ID PXD076844. The corresponding author will provide the data sets and protocols to other investigators upon reasonable request.

## Abbreviations:

ACAA1: acetyl-CoA acyltransferase 1; ACOX1: acyl-CoA oxidase 1; CAT: catalase; CPT1: carnitine palmitoyltransferase 1; CPT2: carnitine palmitoyltransferase 2; DAG: diacylglycerol; DCF: 2′,7′-dichlorodihydrofluorescein; FAO: fatty acid β-oxidation; FFA: free fatty acid; GPCR: G protein-coupled receptor; Hcy: homocysteine; HHcy: hyperhomocysteinemia; LCFA: long-chain fatty acid; LPC: lysophosphatidylcholine; LPE: lysophosphatidylethanolamine; mtROS: mitochondrial reactive oxygen species; PC: phosphatidylcholine; PCA: principal component analysis; PE: phosphatidylethanolamine; PG: phosphatidylglycerol; PRDX1: peroxiredoxin 1; PPARα: peroxisome proliferator-activated receptor alpha; PS: phosphatidylserine; ROS: reactive oxygen species; SCP2: sterol carrier protein 2; SM: sphingomyelin; SOD1: superoxide dismutase 1; TAG: triacylglycerol; TMRM: tetramethylrhodamine methyl ester; TXA2: thromboxane A2; VLCFA: very long-chain fatty acid; ΔΨm: mitochondrial membrane potential.
