# Supplementary material for: Fenofibrate attenuates hyperhomocysteinemia-potentiated thrombosis by restoring platelet fatty acid β-oxidation
Source: Redox Biol. 2026 Jun 6;95:104250. doi: 10.1016/j.redox.2026.104250 (PMC13264288; doi:10.1016/j.redox.2026.104250)
Supplement: Multimedia component 1 [file mmc1.pdf]

Title: Fenofibrate Attenuates Hyperhomocysteinemia-potentiated Thrombosis by Restoring Platelet Fatty Acid β-Oxidation

Authors:

Lulu Han[1, 2#], Xing Du[2#], Yu Yan[2], Linqi Zhang[2], Juan Feng[2*], Xian Wang[2*] and Xingzhong Zhang[2, 3*]

Affiliations

[1]Cancer Institute, Cellular Therapeutics School of Medicine, Xuzhou Medical University, Xuzhou, China.

[2]Department of Physiology and Pathophysiology, School of Basic Medical Sciences, State Key Laboratory of Vascular Homeostasis and Remodeling, Peking University, Beijing, China.

[3]State Key Laboratory of Cardiovascular Disease, Fuwai Hospital, National Center for Cardiovascular Diseases, Chinese Academy of Medical Sciences and Peking Union Medical College, Beijing, China

[#]Lulu Han and Xing Du contributed equally to this work.

*Address correspondence to:

Xingzhong Zhang, E-mail: zhangxingzhong@fuwai.com

Xian Wang, Email: xwang@bjmu.edu.cn

Juan Feng, E-mail: juanfeng@bjmu.edu.cn

Inventory of Supplemental Information

1. Supplemental Methods
2. Supplemental Figures

1. Supplemental Methods

1.1. Lipidomics and Proteomics

Lipid Extraction

Lipids were extracted from approximately 100 million platelets using a modified Bligh and Dyer method[1]. Briefly, cells were homogenized in 750 μL of chloroform/methanol/MilliQ water (3:6:1, v/v/v). The homogenate was incubated for 1 hour at 4°C with constant agitation at 1500 rpm. Phase separation was induced by adding 350 μL of deionized water and 250 μL of chloroform. After centrifugation, the lower organic phase containing the lipids was collected. To maximize lipid recovery, the residual aqueous phase and cell debris were subjected to a second extraction with 450 μL of chloroform. The combined organic extracts were pooled and dried in a SpeedVac concentrator operating in OH mode. The dried lipid samples were stored at -80°C until analysis. The remaining upper aqueous phase and the protein pellet were separately dried in the SpeedVac using H_2O mode. Total protein content was determined from the dried pellet with the Pierce® BCA Protein Assay Kit according to the manufacturer's protocol.

Lipidomics Analysis

Comprehensive lipidomic profiling was performed by LipidALL Technologies using a Shimadzu ExionLC-AD system coupled to a Sciex QTRAP 6500 PLUS mass spectrometer, following an established methodology[2]. Polar lipid classes were separated by normal-phase high-performance liquid chromatography (NP-HPLC) on a TUP-HB silica column (150 mm × 2.1 mm i.d., 3 μm particle size). The mobile phases consisted of (A) chloroform/methanol/ammonium hydroxide (89.5:10:0.5, v/v/v) and (B) chloroform/methanol/ammonium hydroxide/water (55:39:0.5:5.5, v/v/v/v). Data acquisition in multiple reaction monitoring (MRM) mode enabled the comparative profiling of diverse polar lipids. Individual lipid species were quantified by referencing a cocktail of spiked internal standards, which included d9-PC32:0 (16:0/16:0), d9-PC36:1p (18:0p/18:1), d7-PE33:1 (15:0/18:1), d9-PE36:1p (18:0p/18:1), d31-PS (d31-16:0/18:1), d7-PA33:1 (15:0/18:1), d7-PG33:1 (15:0/18:1), d7-PI33:1 (15:0/18:1), d5-CL72:8 (18:2)4, Cer d18:1/15:0-d7, C12:0 Cer-1-P, d9-SM d18:1/18:1, C8-GluCer, C8-GalCer, d3-LacCer d18:1/16:0, Gb3 d18:1/17:0, d7-LPC18:1, d7-LPE18:1, C17-LPI,

C17-LPA, C17-LPS, C17-LPG, and d17:1 Sph (all from Avanti Polar Lipids). GM3-d18:1/18:0-d3 was sourced from Matreya LLC. Free fatty acids were quantified using d31-16:0 (Sigma-Aldrich) and d8-20:4 (Cayman Chemicals) as internal standards.

Glycerolipids, including diacylglycerols (DAG) and triacylglycerols (TAG), were analyzed via a modified reverse-phase HPLC/MRM method[3]. Separation was performed on a Phenomenex Kinetex C18 column (4.6 × 100 mm, 2.6 μm) with an isocratic mobile phase of chloroform/methanol/0.1 M ammonium acetate (100:100:4, v/v/v) at a flow rate of 300 μL/min for 10 minutes. Short-, medium-, and long-chain TAGs were quantified based on the internal standards TAG (14:0)3-d5, TAG (16:0)3-d5, and TAG (18:0)3-d5 (CDN Isotopes), respectively. DAG species were quantified using d5-DAG17:0/17:0 and d5-DAG18:1/18:1 (Avanti Polar Lipids) as standards.

Free cholesterol and cholesteryl esters were analyzed using atmospheric pressure chemical ionization (APCI) on a Jasper HPLC system coupled to a Sciex 4500 MD mass spectrometer, as described previously [4]. Quantification relied on the internal standards d6-cholesterol and d6-C18:0 cholesteryl ester (CE) (CDN Isotopes).

Proteomics Analysis

Proteomic profiling was performed on a Dionex Ultimate 3000 nanoLC system coupled online to a Q Exactive™ HF Hybrid Quadrupole-Orbitrap mass spectrometer (Thermo Scientific). Peptides were separated using a reversed-phase PepMap™ RSLC C18 column (75 μm × 25 cm, 2 μm) with a 120 min linear gradient from 4% to 28% mobile phase B (0.1% formic acid in 80% acetonitrile) at a flow rate of 300 nL/min, while mobile phase A consisted of 0.1% formic acid in water.

Mass spectrometry data were acquired in data-dependent acquisition (DDA) mode, with full MS scans (350 - 1500 m/z) collected at a resolution of 60,000, an AGC target of 3e6, and a maximum injection time of 50 ms. The 15 most intense precursors were sequentially isolated for HCD fragmentation at 28% normalized collision energy. MS/MS scans were recorded at a resolution of 15,000 (AGC target 1e5, maximum injection time 50 ms), applying a dynamic exclusion window of 30 s.

We processed the data with MaxQuant (v2.1.0.0) using the human UniProt database for searches. Carbamidomethylation of cysteine was specified as a fixed modification, and oxidation of methionine and N-terminal acetylation were included as

variable modifications. A false discovery rate (FDR) threshold of 1% was applied at both the peptide and protein levels. Label-free quantification was subsequently performed using the integrated MaxLFQ algorithm.

1.2. Western blot analysis

For the Western blot analysis, washed platelets obtained from C57BL/6J mice were stimulated with thrombin (0.01 U/mL) in the presence or absence of Hcy (100 μM). Post-stimulation, platelets were immediately lysed using RIPA lysis buffer (Beyotime Biotechnology, NJ, CN), supplemented with a comprehensive protease inhibitor cocktail (Targetmol Corp, MA, US) to inhibit aspartyl, cysteine, serine proteases, and aminopeptidases. Following protein purification, samples were separated by SDS-PAGE and subsequently transferred to nitrocellulose membranes via a wet electrophoretic method. The membranes were blocked and then probed overnight at 4°C with specific primary antibodies. The antibodies used targeted phospho-AKT^{Ser473}, phospho-SRC^{Tyr416}, total AKT, total SRC (all from Cell Signaling Technology), PPARα, ACOX1, CPT1a (all from Santa Cruz Biotechnology, CA, USA) and β-actin (ABclonal Biotechnology, Wuhan, CN) for normalization.

For proteomic analysis, 50 μg of protein per sample was reduced with 10 mM dithiothreitol (56°C, 30 min), alkylated with 20 mM iodoacetamide (room temperature, 30 min in the dark), and digested overnight at 37°C with sequencing-grade trypsin at a 1:50 (w/w) enzyme-to-protein ratio using a filter-aided sample preparation (FASP) protocol. Peptides were desalted using C18 StageTips, dried in a vacuum concentrator, and stored at -80°C until LC-MS/MS analysis.

1.3. Mitochondrial ROS and total ROS

Platelets (1×10^7/mL) were incubated with 100 μM Hcy for indicated times at 37°C. The platelets were then stained with 1 μM MitoSOX or 1 μM H2DCFDA (MCE) for 10 minutes at room temperature. After washing with PBS, fluorescence intensity was measured using a Cytation 3 Cell Imaging Multi-Mode Reader (BioTek).

1.4. Flow cytometry analysis

To measure surface expression of P-selectin (CD62P), ROS generation, or mitochondrial membrane potential, washed murine C57BL/6J platelets were adjusted to 1×10^7/mL in modified Tyrode's buffer and stained with saturating concentrations

of FITC-conjugated mouse anti-P-selectin antibody (1:20, Emfret Analytics, DE), 1 μM H2DCFDA, or JC-1 (1 μg/mL, MCE) for 15 minutes at room temperature. The platelets were then stimulated for 20 minutes at 37°C with thrombin (0.01 U/mL) in the presence or absence of Hcy, fenofibrate, AC, or perhexiline. Reactions were stopped by fixation in 1% formaldehyde with 5% BSA, and samples were analyzed immediately using a FACSCalibur flow cytometer (BD Biosciences).

2. Supplemental Figures

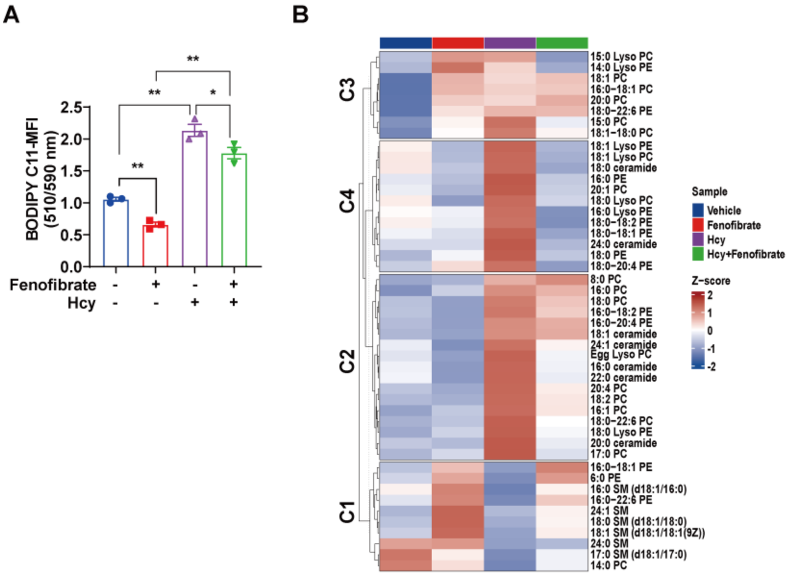

Figure S1. Activation of FAO by fenofibrate remodels platelet lipid metabolism and alleviates mitochondrial dysfunction. (A) Bar graph showing normalized BODIPY 493/503 (neutral lipid-specific probe) MFI-neutral lipid in washed platelets from C57BL/6J mice treated with vehicle (saline), fenofibrate, homocysteine (100 μM) or Hcy + fenofibrate (20 μM). (B) The heatmap depicts the phospholipid metabolic profiles of washed platelets following a 10-minute pre-incubation with vehicle (saline), fenofibrate, homocysteine (Hcy, 100 μM), or Hcy (100 μM) combined with fenofibrate (20 μM) at 37°C, prior to stimulation with the indicated thrombin concentrations. All data are expressed as mean ± SEM; n = 3, **P* < 0.05, ***P* < 0.01.

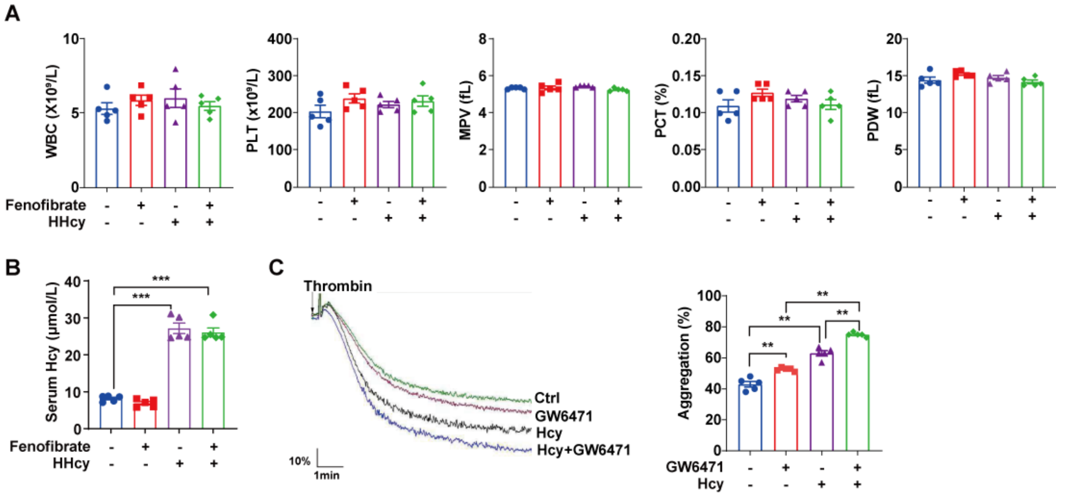

Figure S2. Fenofibrate ameliorates HHcy-induced platelet hyperactivation and reduces thrombus formation. (A) C57BL/6J mice were provided with drinking water containing 1.8 g/L Hcy or plain water for three weeks; after the first week, they received daily administrations of either fenofibrate (100 mg/kg) or vehicle.White blood cell count (WBC), platelet count (PLT), mean platelet volume (MPV), plateletcrit (PCT), and platelet distribution width (PDW) were measured. (B) Serum Hcy levels were determined using ELISA. (C) Platelet aggregation was monitored turbidimetrically with 1 μM GW6471 in the presence or absence of Hcy stimulation with thrombin (0.01 U/mL). All data are expressed as the mean ± SEM. n = 5, ***P* < 0.01, ****P* < 0.001.

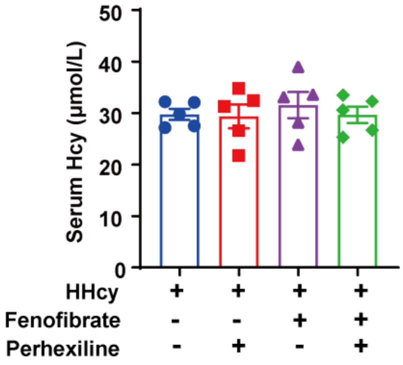

Figure S3. Serum was isolated from the mice in result 5. Hcy levels were subsequently measured via ELISA.

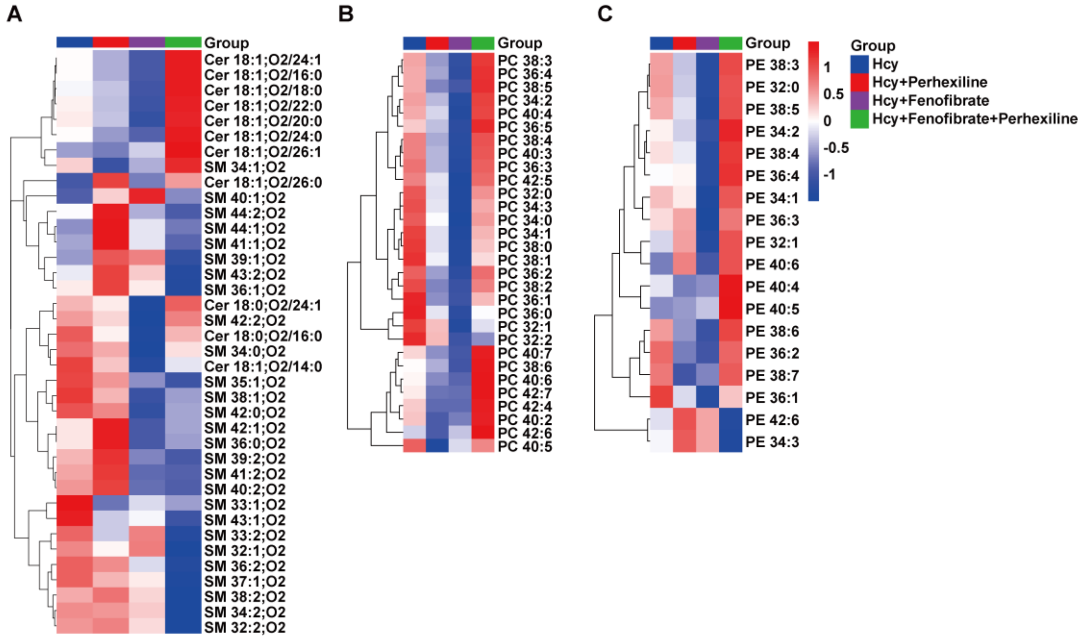

Figure S4. CPT1/2 inhibition by perhexiline reverses HHcy+fenofibrate-induced remodeling of platelet FAO and phospholipids. (A-C) Platelets were isolated from the mice in result 5 for lipidomics analysis. (A) Heatmap displaying the normalized relative levels of individual ceramide and SM species relative to the HHcy group, with color intensity indicating fold-change. (B) Heatmap displaying the normalized relative levels of individual PC species relative to the HHcy group, with color intensity indicating fold-change. (C) Heatmap displaying the normalized relative levels of individual PE species relative to the HHcy group, with color intensity indicating fold-change (red: upregulation, blue: downregulation).

Reference

1. Song JW, Lam SM, Fan X, et al. Omics-Driven Systems Interrogation of Metabolic Dysregulation in COVID-19 Pathogenesis. *Cell Metab*. 2020;32(2):188-202 e185.
2. Lam SM, Zhang C, Wang Z, et al. A multi-omics investigation of the composition and function of extracellular vesicles along the temporal trajectory of COVID-19. *Nat Metab*. 2021;3(7):909-922.
3. Shui G, Guan XL, Low CP, et al. Toward one step analysis of cellular lipidomes using liquid chromatography coupled with mass spectrometry: application to Saccharomyces cerevisiae and Schizosaccharomyces pombe lipidomics. *Mol Biosyst*. 2010;6(6):1008-1017.
4. Shui G, Cheong WF, Jappar IA, et al. Derivatization-independent cholesterol analysis in crude lipid extracts by liquid chromatography/mass spectrometry: applications to a rabbit model for atherosclerosis. *J Chromatogr A*.

2011;1218(28):4357-4365.
